# Effects of short‐lasting supramaximal‐intensity exercise on diet‐induced increase in oxygen uptake

**DOI:** 10.14814/phy2.13506

**Published:** 2017-11-20

**Authors:** Katsunori Tsuji, Yuzhong Xu, Xin Liu, Izumi Tabata

**Affiliations:** ^1^ Faculty of Sport and Health Science Ritsumeikan University 1‐1‐1 Noji‐Higashi Kusatsu City, Shiga Prefecture Japan

**Keywords:** Metabolic chamber, EPOC, diet‐induced thermogenesis, cardiorespiratory fitness

## Abstract

This study was undertaken to quantify the additional increase in diet‐induced oxygen uptake after exhaustive high‐intensity intermittent exercise (HIIE), consisting of 6–7 bouts of 20‐sec bicycle exercise (intensity: 170% $\dot{V}O_{2\max}$) with a 10‐sec rest between bouts. Using a metabolic chamber, the oxygen uptake of ten men was measured from 10:30 am to 07:00 am the next day on two separate days with or without HIIE, with lunch (12:00) and supper (18:00) (Diet experiment). On two other days, the oxygen uptake of six different subjects was measured from 10:30 to 16:00 with or without HIIE, but without meals (Fasting experiment). Ten minutes of exercise at 50% $\dot{V}O_{2\max}$preceded the HIIE in both experiments; EPOC (excess postexercise oxygen consumption) after HIIE was found to wear off before 12:00 in both experiments. In the Diet experiment, oxygen uptake during HIIE and EPOC were 123.4 ± 12.0 and 115.3 ± 32.3 mL·kg^−1^, respectively. Meals elevated resting oxygen uptake on both days, but those on the HIIE day were significantly higher than on the control day. This enhanced diet‐induced oxygen uptake (difference in resting oxygen uptake from 12:00–23:00 between HIIE and control day: ΔDIT) was 146.1 ± 90.9 mL·kg^−1^, comparable to the oxygen uptake during the HIIE and EPOC. The ΔDIT was correlated with subjects’ $\dot{V}O_{2\max}$(52.1 ± 6.6 mL·kg^−1^·min^−1^) (*r* = 0.76, *n* = 10, *P* < 0.05). We concluded that HIIE enhances diet‐induced oxygen uptake significantly, and that it is related to the cardiorespiratory fitness.

## Introduction

Total daily oxygen uptake consists of four components: basal oxygen uptake, diet‐induced oxygen uptake, oxygen uptake during physical activity, and excess‐post exercise oxygen uptake (EPOC).

To elucidate the metabolic effects of physical activity/exercise on energy consumption, the effects of various consumption factors have been studied. Specifically, attempts have been made to study the possible effects of exercise on thermic effects following meals, but have resulted in conflicting conclusions. Bahr and Sejersted (1991) reported that a meal consumed 2 h after 80‐min exhaustive exercise at 75% $\dot{V}O_{2\max}$did not alter oxygen uptake measured during the 7‐h period after the end of the exercise, and concluded that there was no major interaction effect between food intake and exercise on postexercise O_2_ consumption. Ohnaka et al. (1998) also reported that 1 h of bicycle ergometer exercise at 58% $\dot{V}O_{2\max}$ did not affect diet‐induced increase in resting oxygen uptake.

On the other hand, Weststrate and Hautvast (1990) and Young et al. (2010) concluded that intense prolonged prior exercise increased energy expenditure in the postprandial phase. However, reports studying the effects of prior exercise on diet‐induced oxygen uptake have been limited to low‐ and vigorous‐intensity exercise. Only one paper has measured resting oxygen uptake after sprint‐interval exercise using a metabolic chamber, as in our case; that study examined the effect over a 22‐h period, including three meals (Sevits et al. 2013). It was found that there were no observable differences in total resting oxygen uptake during the late recovery phase (3–22 h after the exercise period, at which point the EPOC had worn off) between exercise and nonexercise controls days.

Moreover, Hazell et al. (2012) reported that total oxygen uptake during a 2‐min sprint interval exercise and measured for up to 7 h after exercise was significantly less than that observed during a 30‐min moderate‐intensity exercise, and for the same period thereafter. Meanwhile, the accumulated 24‐h oxygen uptake from the start of both exercises did not differ between the two experiments, suggesting that oxygen uptake during the late recovery period including three meals may have been stimulated by the sprint‐interval exercise. However, this previous paper did not refer to prior high‐intensity exercise‐induced increases in diet‐induced oxygen uptake to explain the increase in resting oxygen uptake during the late period after the sprint‐interval exercise. The result of the Hazell et al. (2012) study suggests that supramaximal‐intensity exercise may elevate diet‐induced oxygen uptake, resulting in increased oxygen uptake during the later phase of rest after exercise.

Treadway and Young (1990) reported that, of exercises performed at 34%, 54%, and 75% $\dot{V}O_{2\max}$, a significant elevation of 100‐g glucose‐induced thermogenesis was observed only after the highest intensity (75% $\dot{V}O_{2\max}$) exercise, suggesting that elevation of glucose metabolism after glucose load is limited to high‐intensity exercise. Furthermore, Denzer and Young (2003) reported that a single bout of high‐intensity resistance exercise enhanced the thermic effect of a carbohydrate meal. Therefore, the purpose of the present investigation was to determine the effect of short‐duration supramaximal‐intensity exercise on diet‐induced increase in oxygen uptake, using a metabolic chamber able to measure oxygen uptake continuously.

The second purpose of this study was to correlate this possible prior‐supramaximal‐intensity‐exercise‐induced increase in diet‐induced oxygen uptake to aerobic fitness. Sato et al. (1986) reported that resting glucose metabolic rate during the euglycemic physiologically hyperinsulinemic condition, which may influence glucose uptake when blood concentrations of glucose and insulin are high after a meal, was relatively highly correlated with the $\dot{V}O_{2\max}$ of their subjects. Furthermore, Burke et al. (1993) reported that the thermic effect of a meal was higher in the group of subjects with high aerobic fitness. These previous studies suggested that the thermic effect of food is augmented in fit individuals. However, no previous studies have reported the effect of aerobic fitness on additional increase in diet‐induced oxygen uptake after supramaximal intensity exercise.

Decreased aerobic fitness is known to be a potent risk factor and predictor of most noncommunicative diseases, for example, diabetes and cardiovascular diseases (Sawada et al. 2003). If the relationship between aerobic fitness and additional increase in diet‐induced resting oxygen uptake after HIIE is to be elucidated, the beneficial effects of improving aerobic fitness should be further emphasized in terms of increasing diet‐induced energy consumption after HIIE. Therefore, we observed the relationship between $\dot{V}O_{2\max}$ and the prior‐supramaximal‐exercise‐induced increase in diet‐induced oxygen uptake.

## Materials and Methods

The present investigation consisted of two main sets of experiments, Diet and Fasting, using a metabolic chamber to measure oxygen uptake after high‐intensity intermittent exercises (HIIE).

The order of exercise versus nonexercise control experiments was randomized among subjects, and experiments on the same subject were separated by at least a week. All exercise was performed using a mechanically braked cycle ergometer (Ergomedic 828E, Monark, Stockholm, Sweden).

The protocols for the experiments and procedures involved in the present investigation were approved by the Ethics Committee of Ritsumeikan University. Subjects were recruited at Ritsumeikan University by a recruiting message posted on a laboratory homepage operated by the authors. After receiving a detailed explanation of the purpose, potential benefits, and risks of participating in the study, each subject gave written informed consent. Subjects were excluded from this study if they had evidence of cardiovascular diseases, anemia, diabetes, renal or hepatic diseases, hypo‐ or hyper‐thyroidism, or musculoskeletal problems. Smokers, and persons taking medications were also excluded. The subjects were told not to alter their dietary and exercise habits during the experimental period.

### Pretest

All exercises were conducted on a mechanically braked cycle ergometer at 90 repetitions per minute (rpm). Since exercise intensity used in the present investigation is expressed relative to the $\dot{V}O_{2\max}$, that value was established on pretests as follows. First, to determine a linear relationship between the submaximal intensity of bicycling (watts) and the steady‐state oxygen uptake (L·min^−1^) for each individual, oxygen uptake was measured during the last 2 min of six to nine different 10‐min bouts of bicycling at a constant power between 35 and 90% $\dot{V}O_{2\max}$. Next, to determine $\dot{V}O_{2\max}$, oxygen uptake was measured during the last two or three 30‐sec intervals during several bouts of supramaximal‐intensity exercises that exhausted subjects within 2–4 min. After confirming a leveling‐off in oxygen uptake by increasing intensity, the highest oxygen uptake measured was taken as the subject's $\dot{V}O_{2\max}$ (Taylor et al. 1955; Medbø et al. 1988; Tabata et al. 1996; Matsuo et al. 2017).

To determine 170% $\dot{V}O_{2\max}$ and the corresponding bicycling exercise intensity, using the established relationship between power and steady‐state oxygen uptake described above, a linear extrapolation to higher powers was carried out. An oxygen demand of 170% $\dot{V}O_{2\max}$ was taken as 1.70 times of $\dot{V}O_{2\max}$ (L·min^−1^), and the corresponding biking power was determined from the linear relationship (see Figure 1 in Tabata et al. (1997)).

### Diet experiments

Ten young male subjects volunteered for this experiment. Their age (years), height (m), body mass (kg), body mass index (BMI), and $\dot{V}O_{2\max}$ (mL kg^−1^ min^−1^) were 23 ± 1, 1.71 ± 0.05, 64.4 ± 6.0, 22.1 ± 1.7, and 52.1 ± 6.6, respectively (means ± SDs).

The subjects refrained from any structured exercise on the day before each experiment. Subjects in both experiments were also instructed to eat their usual meals on the day prior to the experiment. They were asked to record these meals, but were not required to report in detail (i.e., grams of all food items) the content of meals ingested on the day before the first chamber experiment. An experienced registered dietitian ensured that the meals consumed by the individual subjects were adequate for Japanese individuals in terms of a food intake pattern that reflects calorie, carbohydrate, fat, and protein intake. No alcohol or caffeine was allowed in the 24 h before either experiment. These instructions were consistent for all experiments (i.e., the Diet and Fasting experiments).

Several days before the first chamber experiment, subjects were invited to come to the chamber and stay for a couple of hours so that they could become familiar with it. On the experiment day, subjects ate breakfast at 08:00 (Table 1, left panel). Just before each subject entered the metabolic chamber at 10:00, he put on a mask for expired gas collection by the Douglas bag method. After the subject entered the chamber, the door to the chamber was closed, and the subject himself connected his mask to a hose; the end of the hose was connected to a three‐way cock located outside the chamber. The length of the hose was 1.0 m.

**Table 1:** Timetable of Diet experiments and Fasting experiments

Diet experiments (*n* = 10)		Fasting experiments (*n* = 6)	
7:00	Get up	7:00	Get up
**8:00‐8:20**	**Breakfast**	**8:00‐8:20**	**Breakfast**
8:20‐10:00	Sit quietly	8:20‐10:00	Sit quietly
10:00‐10:10	Rest on the bicycle ergometer	10:00‐10:10	Rest on the bicycle ergometer
10:10‐10:20	Warm up	10:10‐10:20	Warm up
10:20‐10:30	Rest on the bicycle ergometer	10:20‐10:30	Rest on the bicycle ergometer
**10:30**	**HIIE or Rest**	**10:30**	**HIIE or Rest**
After exercise ‐10:48	Rest on the bicycle ergometer	After exercise ‐10:48	Rest on the bicycle ergometer
10:48‐12:00	Repeat	10:48‐16:00	
	10‐min Desk work		Repeat
	20‐min Lie awake in supine position		10‐min Desk work
**12:00‐12:20**	**Lunch**		20‐min Lie awake in supine position
12:20‐18:00	Repeat		
	10‐min Desk work		
	20‐min Lie awake in supine position		
**18:00‐18:20**	**Supper**		
18:20‐23:00	Repeat		
	10‐min Desk work		
	20‐min Lie awake in supine position		
23:00	Sleep to the next morning		
Next morning			
7:00	Get up		

Once the subject was inside the chamber, the operation for measuring oxygen consumption was initiated. Since each subject wore a mask, all expired gas was collected and sent outside the chamber, so that there was no trace of oxygen consumption in the chamber after the first minutes. Outside of the chamber, the tester manipulated the three‐way cock as for an ordinary expired‐gas measurement in a laboratory.

Twenty minutes before the subjects started HIIE at 10:30, they biked for 10 min at 50% $\dot{V}O_{2\max}$ intensity as a warm‐up exercise. The HIIE that subjects performed in the metabolic chamber is an exhaustive exercise consisting of 6–7 bouts of 20‐sec high‐intensity intermittent bicycle exercise with 10‐sec rests between bouts. The exercise intensity was 170% $\dot{V}O_{2\max}$; 6–7 bouts at this level have been shown to exhaust a subject (Tabata et al. 1997). This HIIE protocol has been shown to stimulate both aerobic and anaerobic energy‐releasing systems maximally, and to improve both aerobic and anaerobic energy‐releasing systems simultaneously (Tabata et al. 1996; Tabata et al. 1997). The subjects continued to wear the mask and sit on the bicycle ergometer for 15 min after the end of the HIIE. Subjects then removed the mask themselves. Through a glass window, a tester outside the metabolic chamber monitored the workload continuously and gave verbal instructions through a microphone with a speaker inside the chamber. This was to ensure that the work output was precisely the same as those done in experiments conducted in ordinary experimental rooms.

The subjects then alternated between two activities – 20 min of lying on the bed awake, and 10 min of sitting on a chair doing deskwork – until 23:00, when the subjects were allowed to sleep. During this time, subjects read books or studied. They were not allowed to watch DVDs or videos. They got up and left the chamber at 07:00 the next morning.

Lunch and supper were served at 12:00 and 18:00. The meals were prepared based on the estimated energy requirement for Japanese subjects (Ministry of Health, Labour and Welfare of Japan, Dietary Reference Intakes for Japanese, 2010 assuming a physical activity level of 1.5. The mean energy content of lunch and supper during the experimental day was 2186 kcal/day. The meals contained 15% protein, 25% fat, and 60% carbohydrate in energy equivalents, which is the mean value for the Japanese population (Dietary Reference Intakes for Japanese, 2010; http://www.nibiohn.go.jp/eiken/info/pdf/dris2010en.pdf). The subjects ate full meals, and were allowed to drink water ad libitum during their stay in the chamber.

For the nonexercise control experiment, the subjects followed the same protocol as in the HIIE experiment until the next morning, but did not perform the HIIE.

### Fasting experiments

The purpose of this experiment was to make sure that the EPOC of the preceding supramaximal‐intensity exercise had worn off before subjects ate lunch in the Diet experiments. Six different young male subjects than those enrolled in the Diet Experiments, volunteered for this experiment. Their age (years), height (m), body mass (kg), BMI, and $\dot{V}O_{2\max}$ (mL·kg^−1^·min^−1^) were 22 ± 0, 1.77 ± 0.06, 68.8 ± 6.7, 21.8 ± 1.4, and 53.2 ± 5.3, respectively (means ± SDs).

On the HIIE experiment day, the subjects entered the metabolic chamber at 10:00 after eating breakfast at 8:00 (Table 1, right panel). They started the HIIE at 10:30 following the same protocol as in the Diet Experiments. The only difference was that they were not served lunch. Otherwise the subjects followed the same protocol as on the exercise day of the Diet Experiments, until 16:00, when they left the chamber.

For the nonexercise control experiment day, the subjects followed the same protocol as on the HIIE experiment day until 16:00, except that they did not perform the HIIE.

### Oxygen uptake measurement using douglas bag

Fractions of oxygen and carbon dioxide in the expired air were measured by a mass spectrometer (Arco 2000; Arcosystems, Kashiwa, Chiba, Japan). Gas volume was measured by a gasometer (Shinagawa Seisakusho, Shinagawa, Tokyo, Japan).

The reason why we used the Douglas bag method for this “metabolic chamber HIIE study” was to avoid the assumed artifact effects of HIIE on calculated oxygen uptake during and after the HIIE, which induces sudden and rapid increases in oxygen uptake and carbon dioxide production that may interfere with the calculation of oxygen consumption using the modified Henning equation (Henning et al. 1996; Nguyen et al. 2003).

For the Diet Experiments, the calculated oxygen uptake during the first minute (i.e., 15–16 min after the HIIE) using the chamber (0.425 ± 0.060 L·min^−1^) was comparable to the measured oxygen consumption during the last minute (i.e., 14–15 min after cessation of the HIIE) using the Douglas bag method (0.433 ± 0.061 L·min^−1^), demonstrating the concurrence of oxygen uptake measurement between the Douglas bag method and the metabolic chamber measurement.

### Metabolic chamber

An open‐circuit indirect metabolic chamber (Fuji Human Calorimeter, Fuji Ika Sangyo, Chiba, Japan) was used to measure oxygen uptake for this study. The temperature and relative humidity in the room were controlled at 25°C and 50%, respectively; the internal area and volume of the chamber were 8.1 m^2^ and 18.5 m^3^, respectively. Fans were used to move air around the room. The chamber was a pull calorimeter (i.e., the flow was controlled and measured at the outlet at a rate of 80 L·min^−1^). To measure the outgoing flow, the chamber was equipped with a mass flow controller (CMQ02, Yamatake, Tokyo, Japan). Concentrations of gases in the outgoing air were measured with high precision by online process mass spectrometry (VG Prima dB; Thermo Fisher Scientific, Winsford, U.K.). The mass spectrometer measured fractional concentrations of O_2_, CO_2_, N_2_, and Ar. Oxygen uptake was calculated by utilizing a modified Henning equation (Henning et al. 1996; Nguyen et al. 2003). The accuracy and precision of the metabolic chamber for measuring oxygen uptake, as determined by the alcohol combustion test, was 100.6 ± 0.9% (mean ± SD) over 3 h. The concentrations of the gases in the outside air were measured every hour to evaluate any effects of the changed concentration of incoming gases on calculated oxygen uptake.

#### Statistical analysis

Values are shown as means ± SDs. The data were analyzed using repeated‐measures two‐way analysis of variance (ANOVA) to determine the degree of significance of among‐group differences. The significance level for all comparisons was set at *P* < 0.05.

Required sample sizes to detect the HIIE effect on ΔDIT in the Diet Experiments and EPOC in the Fasting Experiments were 10 and 6, respectively (G* Power ver. 3.1 (SOFTPEDIA) (*α* = 0.05 and power = 0.8).

## Results

### Diet experiments

**Figure 1:**
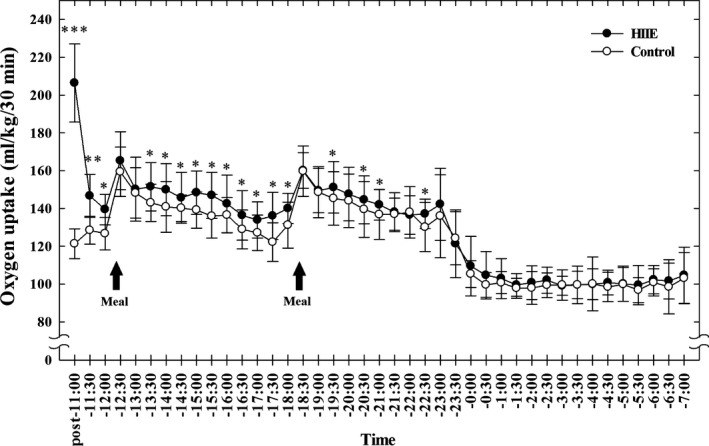
Resting oxygen uptake measured from the end of the HIIE to 07:00 on the next day in the Diet Experiments. Values are mean±SD (*n* = 10). *P < 0.05, **P < 0.01, ***P < 0.001 between the HIIE day and the nonexercise control day.

Average exercise time of subjects was 138.9 ± 10.5 sec (127–159 sec). Figure 1 shows oxygen uptake measured for every 30 min from the end of HIIE to 07:00 the next morning. Compared with the nonexercise control day, oxygen uptake on the exercise day was significantly higher from the end of the HIIE until 12:00 (*P* < 0.001). No significant differences in oxygen uptake were noted from 12:00 to 13:00, when the subjects ate lunch. Compared with the nonexercise control day, oxygen uptake was significantly higher from 13:00 to the beginning of supper at 18:00 on the HIIE day (*P* < 0.05). Again, no difference in oxygen uptake was observed from 18:00 to 19:00, when supper was served. Furthermore, oxygen uptake was significantly increased from 19:00 to the start of sleep at 23:00 (*P* < 0.05). There was no difference in oxygen uptake between the exercise day and nonexercise day from 23:00 to the next morning at 7:00.

Oxygen uptake during the 10‐min warm‐up exercise was 227.8 ± 25.0 mL·kg^−1^. Meanwhile, oxygen uptake during the HIIE was 123.4 ± 12.0 mL·kg^−1^. The accumulated oxygen uptake (AOU)  – which is defined as the time integration of resting oxygen uptake per body weight between specific time intervals (mL·kg^−1^)  –  from the end of the HIIE to 12:00 on the HIIE day (488.9 ± 35.0 mL·kg^−1^) was significantly higher than on the nonexercise control day (373.6 ± 20.8 mL·kg^−1^) (*P* < 0.001). The AOU from 12:00 to the start of supper at 18:00 on the HIIE day (1714.5 ± 126.9 mL·kg^−1^) was also higher than on the control day (1627.5 ± 108.9 mL·kg^−1^) (*P* < 0.001). Moreover, AOU from 18:00 to the beginning of sleep at 23:00 on the HIIE day (1438.1 ± 105.9 mL·kg^−1^) was higher than on the control day (1379.1 ± 95.2 mL·kg^−1^) (*P* < 0.001), as was AOU from the end of the HIIE to 23:00 (HIIE day: 3641.5 ± 253.0 mL·kg^−1^; control day: 3380.2 ± 214.6 mL·kg^−1^ (*P* < 0.001)). Sleeping oxygen uptakes from 23:00 to 07:00 the next day for the HIIE day (1623.8 ± 132.6 mL·kg^−1^) and control day (1593.4 ± 83.9 mL·kg^−1^) were not significantly different.

The difference in AOU between the HIIE day and nonexercise control day measured from the end of the HIIE to 12:00 (EPOC) was 115.3 ± 32.3 mL·kg^−1^. The total difference of oxygen uptake between the HIIE day and nonexercise control day measured from 12:00 to the start of supper at 18:00 was 87.1 ± 53.3 mL·kg^−1^. The total difference of oxygen uptake between the HIIE day and nonexercise control day measured from 18:00 to the start of sleep at 23:00 was 59.0 ± 65.6 mL·kg^−1^. ΔDIT, which is regarded as the additional DIT due to HIIE, and is calculated as the difference in measured OU from 12:00–23:00 between the exercise day and control day, was 146.1 ± 90.9 mL·kg^−1^. The total difference in AOU between the HIIE day and nonexercise control day measured from the end of the HIIE to 23:00 was 261.4 ± 114.6 mL·kg^−1^.

**Figure 2:**
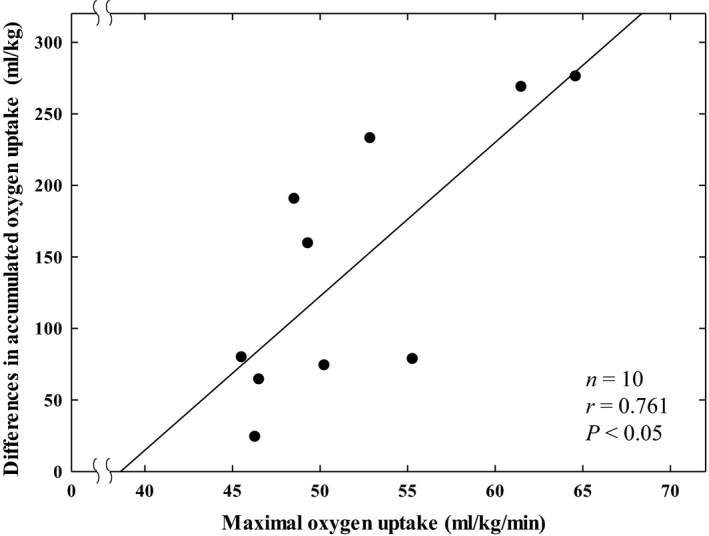
The relationship between differences in oxygen uptake accumulated from the start of lunch (12:00) to the start of sleep (23:00) between the exercise day and control day (mL·kg^−1^) and maximal oxygen uptake (mL·kg^−1^ min^−1^) of the subjects in the Diet Experiments (*n* = 10).

The total difference in oxygen uptake accumulated from the start of lunch to the start of sleep between the exercise day and control day (ΔDIT) was significantly related to the $\dot{V}O_{2\max}$ values of the subjects (*r* = 0.76, *P* < 0.05, *n* = 10, Fig. 2).

**Figure 3:**
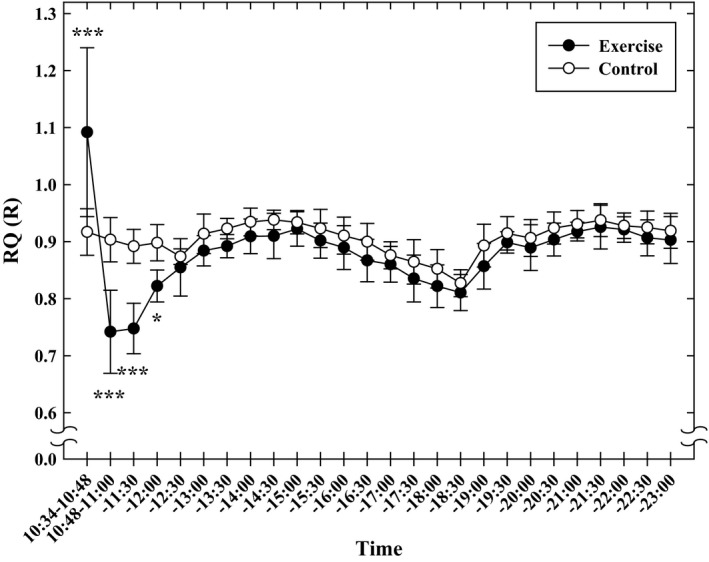
The respiratory quotient (RQ) and respiratory exchange ratio (*R*) measured from the end of the HIIE to 23:00 in the Diet Experiments. Values are mean ± SD (*n* = 10). *P < 0.05, **P < 0.01, ***P < 0.001 between the HIIE day and nonexercise control day.

Figure 3 shows changes in R (respiratory exchange ratio) and RQ (respiratory quotient). R (RQ) on the exercise day was significantly higher from immediately after to 14‐min after the HIIE than on the non‐exercise day (*P* < 0.05). On the contrary, after this time period, R(RQ)s on the exercise day were significantly lower than those observed on the non‐exercise control days until lunch at 12:00 (*P* < 0.05). No significant differences in RQ (R) were noted after this time until bedtime, 23:00, between the two days. On both days, lunch and supper elevated RQ values.

### Fasting experiments

**Figure 4:**
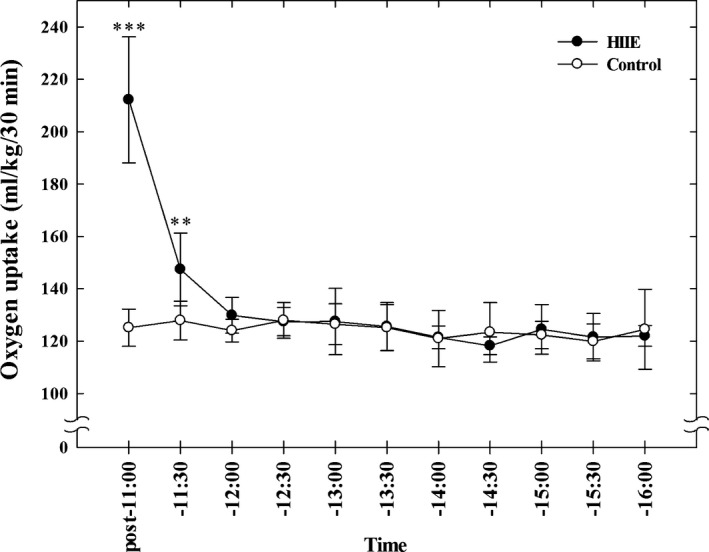
The resting oxygen uptake measured from the end of the HIIE to16:00 in the Fasting Experiments. Values are mean ± SD (*n* = 6). **P < 0.01, ***P < 0.001 between the HIIE day and nonexercise control day.

Average exercise time of subjects was 138.8 ± 8.1 sec (133–155 sec). Figure 4 shows the oxygen uptake for each 30‐min period from the end of the HIIE to 16:00 in the Fasting experiments. Oxygen uptake during the 10‐min warm‐up exercise was 224.2 ± 22.7 mL·kg^−1^; and during the HIIE it was 117.9 ± 17.9 mL·kg^−1^.

Compared with the nonexercise control day, oxygen uptake after HIIE was significantly higher from the end of the HIIE until 11:30 (*P* < 0.001). However, no significant difference in oxygen uptake was observed between the two days from 11:30 to 12:00.

AOU from the end of the HIIE to 16:00 on the HIIE day (1479.6 ± 36.9 mL·kg^−1^) was higher than from the same period on the control day (1358.9 ± 70.5 mL·kg^−1^) (*P* < 0.01). The difference in AOU between the HIIE day and nonexercise control day measured from the end of the HIIE to 11:30 was 115.9 ± 12.8 mL·kg^−1^. The difference in AOU between the HIIE day and nonexercise control day measured from the end of the HIIE to 16:00 was 120.7 ± 50.1 mL·kg^−1^.

**Figure 5:**
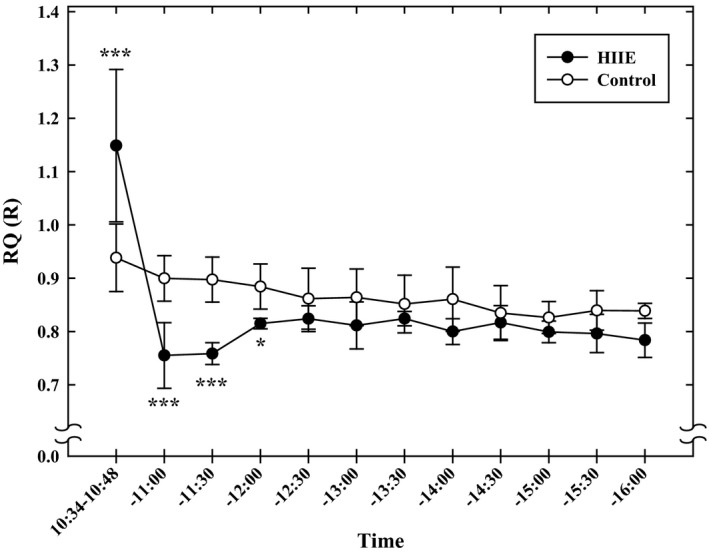
The RQ and R measured from the end of the HIIE to 16:00 in the Fasting Experiments. Values are mean ± SD (*n* = 6). *P < 0.05, ***P < 0.001 between the HIIE day and nonexercise control day.

In the Fasting experiments (Fig. 5), R (RQ) values after HIIE were significantly higher than control day values until 14‐min after the HIIE (*P* < 0.001), and then lower until 12:00 (*P* < 0.001 and *P* < 0.05). After 12:00, no differences in RQ (R) were found between the two days. Furthermore, on both days, RQ (R) did not significantly change until 16:00. In other words, averaged RQ from 12:00–12:30 was not significantly different from that averaged from 15:30–16:00.

## Discussion

The main finding of this study was that HIIE elevated the diet‐induced increase in resting oxygen uptake during the 10.5‐h period after exercise (ΔDIT). ΔDIT was comparable to the resting oxygen uptake values observed during the HIIE, and the EPOC measured during the first 1.5 h after HIIE. Furthermore, the present investigation demonstrated that ΔDIT is significantly related to cardiorespiratory fitness measured as the $\dot{V}O_{2\max}$ of subjects.

Since the Fasting experiments demonstrated that, after 11:30, which corresponded to the time 56 min after exercise, there was no observable difference in resting oxygen uptake between the HIIE day and nonexercise day, the effects of the HIIE on resting oxygen uptake (i.e., EPOC) is regarded to have worn off by this point. EPOC after HIIE was also worn off on the Diet experiments. Generally, the duration of EPOC depends on the intensity and duration of submaximal exercise (Gore and Withers. 1990). For supramaximal‐intensity exercise, Bahr et al. (1992) reported that EPOC lasts for 30 min, 1 h, and 4 h after one, two, and three bouts of 2‐min supramaximal exercise (108% $\dot{V}O_{2\max}$), respectively, suggesting that the persistence of EPOC depends on the number of supramaximal bouts (duration, work) of the exercise. This previous study reported an oxygen deficit, which is the metabolic basis of EPOC for the three exercise sets with varying number of bouts. The oxygen deficit for one, two, and three bouts of the 2‐min exercise in the previous study were 3.2 ± 0.1 L, 5.6 ± 0.2 L, and 7.5 ± 0.2 L (mean ± SE), respectively. The oxygen deficit of the HIIE in the present investigation was calculated to be 4.1 ± 1.2 L for our lighter weight subjects (64.4 ± 6.0 kg vs. 75.0 ± 3.7 kg) (Bahr et al. 1992). Since the oxygen deficit observed in the present investigation was between the values observed after 1 and 2 bouts of the 2‐min exercise of the previous study, which found significant EPOC had worn off at 60 and 90 min after exercise, it is reasonable to assume that significant EPOC had disappeared at this point.

Since EPOC after HIIE was worn off before subjects consumed lunch in the Diet experiments, the difference in resting oxygen uptake after lunch between the exercise and nonexercise day in the Diet experiments could be attributed to diet. Diet‐induced thermogenesis (DIT) itself, and oxygen consumption for DIT, may not have differed between the HIIE day and no‐exercise day in the Diet experiments because the subjects ate the same diet on both days. Davis et al. (1983) reported that DIT was related to $\dot{V}O_{2\max}$(*r* = 0.658, *P* < 0.01, *n* = 26). In accordance with this previous study, the AOU from the start of lunch to the beginning of sleep on the nonexercise control day of the Diet experiments in this investigation was related to $\dot{V}O_{2\max}$ (*r* = 0.69, *P* < 0.05, *n* = 10). Ebeling et al. (1993) showed that the amount of GLUT4 in *m. quadriceps vastus lateralis* was linearly related to $\dot{V}O_{2\max}$ (*r* = 0.61, *P* < 0.04, *n* = 19). This abundance of GLUT4 might be translocated to the plasma membrane of the muscle, possibly by lunch‐induced secretion of insulin in an aerobically fit person, and might consume more oxygen for metabolizing more glucose to glycogen than in an unfit person.

Although the energy intake for lunch (713 kcal) was less than that of supper (919 kcal), the accumulated resting oxygen uptake after lunch (12:00–17:00: 1375.3 ± 93.4 mL/(kg·5 h)) was not significantly different from that after supper (18:00–23:00: 1379.1 ± 95.2 mL/(kg·5 h) on the nonexercise control day. In addition, the ΔDIT measured from 12:00 to 17:00 (71.3 ± 48.2 mL/kg) was not significantly different from that measured from 18:00 to 23:00 (59.0 ± 65.6 mL/kg). These results may indicate that the ΔDIT produced by the HIIE is not related to the ingested energy from the meal on the exercise day. These results might also indicate that ΔDIT is a result of factor(s) that persist for a long time (to 23:00) and gradually wear off during that time period. Increased glucose uptake and/or insulin sensitivity, which last for a long time and wear off gradually (Gulve et al. 1990) are possible candidates that may explain these results.

It has been reported that resting oxygen uptakes during sleep after high‐intensity exercises are not different from those observed after control sedentary activities (Hazell et al. 2012; Sevits et al. 2013; Skelly et al. 2014). In the present investigation, we also observed no difference in the sleeping oxygen uptake between the exercise and control days. The past and present findings taken together suggest that sleeping metabolism may not be elevated by prior high‐intensity intermittent exercise.

Furthermore, the present investigation, demonstrated for the first time that HIIE increases oxygen uptake after lunch and supper to a greater degree than the increases with meals on non‐HIIE control days. Even though the quantity of oxygen and energy consumption (47.8 kcal) was small, it may represent the physiological characteristics of such high‐intensity exercise in terms of energy consumption. Previous studies reported that resting oxygen uptake increased after supramaximal‐intensity exercise. However, since no diet was prepared (Bahr et al. 1992; Burns et al. 2012; Chan and Burns 2013; Skelly et al. 2014; Tucker et al. 2016), it is not possible to study the effects of prior supramaximal exercise on diet‐induced resting oxygen uptake with these papers. Hazell et al. (2012) reported that oxygen uptake measured by the online breath‐by‐breath method during the 30‐min period after eating lunch and dinner was not different from that measured without the 2‐min sprint‐interval exercise. However, that study did not measure oxygen uptake during the 4‐ to 5‐h period after lunch and dinner, when a significant difference in resting oxygen uptake between the exercise day and nonexercise day was observed. Therefore, this previous study may have overlooked prior‐exercise‐induced increase in resting uptake.

Using a hood system for oxygen uptake measurements, Kelly et al. (2013) reported that total EPOCs during the 9.75‐h recovery period starting 1.25 h after two high‐intensity exercise sessions were not different from that measured during the same period on the nonexercise control day. During this period, breakfast, lunch, and dinner were served to subjects. Although this result may be understood to imply that diet‐induced increase in oxygen uptake was not detected even with three meals, this study did not refer to the effects of prior HIIE on diet‐induced increase in oxygen uptake.

The interesting study that is most similar to our present investigation is by Sevits et al. (2013). Using the same methodology (a metabolic chamber), Sevits et al. observed that the oxygen uptake during the 4‐hr period after each of three meals was not different between the day of a sprint‐interval exercise and the nonexercise day. As we mentioned, in this study the diet‐induced oxygen uptake after supramaximal exercise was higher than that measured on the nonexercise control day (Fig. 1). It is not clear why the finding of the two studies differ. First, the EPOC wore off within 2 h after the supramaximal exercise in both studies. The subjects’ age, peak oxygen uptake, BMI, and dietary energy intakes per body weight of the two studies are also similar. The exercise protocol differed somewhat between the studies. In the Sevits et al. study, the subjects exercised for 150 sec (30 sec × 5 times), whereas in our study they exercised from 120 to 140 sec (20 sec × 6 or 7 times). However, the average work (in Joules/body weight) performed during the entire bicycle exercise period in the Sevits et al. study was also similar to that observed herein. The only potentially significant difference is the timing of breakfast and exercise. The subjects in the Sevits et al. study performed supramaximal‐intensity exercise after an overnight fast and before their first meal, whereas in our study, the exercise was performed after breakfast. Regarding timing, the systemic effects of the order of exercise and breakfast (or the fasting state) on the prior exercise‐induced resting oxygen uptake after subsequent meals has not been discussed in the existing literature (Bahr and Sejersted 1991). Further investigations may clarify this issue.

As for absolute volume, the EPOC on the HIIE day (Diet experiments) was 8.0 ± 1.3 L in the present investigation. This value was as high as those observed after a 90‐min cycling exercise at 56% $\dot{V}O_{2\max}$ (8.1 L) (Børsheim et al. 1998) and a 120‐min cycling exercise at 51% $\dot{V}O_{2\max}$ (7.8 L) (Bahr et al. 1987). The absolute volume of EPOC after HIIE in the present investigation appears to be less than that reported for the 23‐h chamber study using a set of 5 maximal 30‐sec bicycle bouts with 3‐min rests between bouts (225 kcal/day) (Sevits et al. 2013). This difference may be explained by the difference in the amount of work (oxygen deficit) executed in the two studies.

Our quantitative analyses of oxygen consumption during and after exercise and diet revealed the unique features of the high‐intensity intermittent exercise. The energy consumption calculated from resting oxygen uptake during the 10‐min warm‐up exercise, HIIE, and EPOC during the first 1.5 h, and ΔDIT after HIIE, were 74.3 ± 5.2, 39.8 ± 6.3, 37.5 ± 12.7, and 47.8 ± 32.0 kcal for our 10 subjects, whose mean weight was 64.4 ± 6.0 kg. ΔDIT was comparable to energy consumption during the HIIE and the EPOC during the first 1.5 h after HIIE. The volume of EPOC has been estimated be ~15% of resting oxygen uptake during exercise (Food and Nutrition Board 2002). However, the present investigation demonstrated that EPOC during the first 1.5 h after HIIE equaled 92.4 ± 18.7% of the resting oxygen uptake during HIIE. Furthermore, the EPOC value after the first 1.5 h and the ΔDIT after HIIE were 60.1 ± 12.8% and 74.0 ± 41.1%, respectively. Summation of these two values amounted to 134.0 ± 47.5% of the accumulated oxygen demand of the HIIE (191.1 ± 25.5 mL/kg)).

In terms of oxygen uptake during exercise, the energy consumption of HIIE seems to be limited. However, EPOC and ΔDIT might have a minimal impact on energy consumption during the training period using HIIE, even though no significant decrease in body weight has been reported after short‐term HIIE training (Tabata et al. 1996). Summation of the previously described four types of energy consumption for subjects weighing 64.4 ± 6.0 kg were 199.4 ± 12.4 kcal, which could have a significant effect on body weight reduction (Hill et al. 2003).

The present investigation, demonstrated for the first time that the differences in oxygen uptake accumulated from the start of lunch to the start of sleep between the supramaximal‐intensity exercise day and control day (ΔDIT) are significantly related to the $\dot{V}O_{2\max}$ values of subjects (Diet Experiments) (*r* = 0.76, *P* < 0.05, *n* = 10, Fig. 2), suggesting that in addition to the stimulating effects of cardiorespiratory fitness on DIT, HIIE further enhances metabolism over DIT. This correlation of two physiological parameters may suggest that this is not a coincidence, but rather a physiologically meaningful relationship. During the relevant time of day (noon to 23:00), the effects of HIIE on resting oxygen consumption were shown to have worn off, and oxygen consumption was supposed to be limited to resynthesizing ATP for resting metabolism and DIT itself, which is assumed to be the same on the two days. Therefore, the higher oxygen uptake on the HIIE day after lunch and supper compared to the nonexercise day could be due to HIIE. The additional oxygen uptake over DIT on the exercise day could be explained by the oxygen consumption due to increased insulin sensitivity by the preceding HIIE. Physical exercise/muscle contraction is known to increase insulin sensitivity, which is defined in terms of the concentration of insulin required to cause 50% of the maximal response. Insulin sensitivity elevation by exercise lasts up to 2 days after exercise (Mikines et al. 1988). Therefore, increased insulin‐stimulated glycogen synthesis by HIIE might cause additional oxygen uptake after lunch and supper on the HIIE day over DIT on the control day. Since the increase in insulin sensitivity is known to be related to the $\dot{V}O_{2\max}$ of subjects (Sato et al. 1986; Larsen et al. 2012), it is reasonable to speculate that the increase in oxygen uptake over DIT‐induced increase in oxygen uptake is related to the $\dot{V}O_{2\max}$ of the subjects in the present investigation.

Our present findings demonstrated that the higher the cardiorespiratory fitness, the higher the diet‐induced energy consumption after high‐intensity intermittent exercise (HIIE). Therefore, HIIE training may contribute to a further increase in energy consumption which may reduce body weight, since such training elevates the $\dot{V}O_{2\max}$ and the elevated $\dot{V}O_{2\max}$ may further increase in diet‐induced energy consumption after HIIE, even if the diet‐induced energy consumption related to $\dot{V}O_{2\max}$ is quantitatively small.

Since R (RQ) is higher than 1.0 during HIIE, and less than 0.7 during the period immediately after HIIE, energy consumption in calories during HIIE and the following recovery period cannot be calculated using oxygen uptake data and RQ or R. This is the case even with currently available formulas such as the Weir method (Weir 1949), which hypothesized that the range of RQ is 0.7–1.0. As one of the purposes of the present investigation was to compare energy consumption during HIIE, the immediate recovery, and the late recovery including DIT, we did not use calories but used AOU as a common measure for evaluating the impact of HIIE on different elements of daily energy consumption. However, since it was considered worthwhile to present calorie values related to HIIE energy consumption in this study, RQ(R) were calculated. Results showed that calorie consumption on the HIIE day from the start of lunch (12:00) to beginning of sleep (23:00) were calculated to be 1012.4 ± 100.9 kcal, while that calculated for the non‐HIIE control day were 964.6 ± 81.8 kcal, indicating that ΔDIT in calories by HIIE was 47.8 ± 32.0 kcal. In addition, calorie consumption during the warm‐up exercises was calculated to be 74.3 ± 5.2 kcal. Calorie consumption for HIIE and EPOC (from the end of HIIE to 12:00) was estimated to be 39.8 ± 6.3, and 37.5 ± 12.7 kcal, respectively; these values were calculated as 1.0 L of oxygen consumption equals 5.0 kcal energy release, because RQ (*R*) values observed at this period did not stay in the 0.7–1.0 range in which energy consumption is calculated using RQ (*R*). Furthermore, ΔDIT in kcal/body weight, calculated using RQ, was confirmed to be correlated with $\dot{V}O_{2\max}$ of the subjects (mL·kg^−1^ min^−1^) (*r* = 0.81, *P* < 0.01), suggesting that high aerobic fitness is related not only to higher AOU, but also to higher thermogenesis induced by diet after HIIE.

Previous studies (Acheson et al. 1984; Berne et al. 1989) suggested that diet, especially high‐carbohydrate meals, induces activation of sympathetic activity, resulting in elevated oxygen consumption/metabolic rate. Therefore, the result that diet induced increased oxygen uptake at rest without HIIE is related to $\dot{V}O_{2\max}$ in the present investigation, and may be explained by enhanced sympathetic activity induced by a mixed meal in aerobically fit individuals (Young et al. 2010) and/or the greater adrenergic thermogenic responsiveness of habitually exercising adults (Stob et al. 2007). Furthermore, it is possible that the high‐carbohydrate diets enhanced a sympathetic nervous system already activated by HIIE. However, since no reports are available regarding the effects of prior‐exercise on diet‐induced sympathetic nervous activity, it is not feasible for us here to propose plausible mechanisms explaining the additional increase in oxygen uptake after meals followed by HIIE. Future research, for example, on the effects of *β*‐agonist (isoproterenol) infusion on oxygen uptake after meals followed by HIIE, will be necessary to clarify the mechanisms of this result.

Laforgia et al. (1997) reported that after supramaximal intensity intermittent exercise (ten 1‐min exercise at 105% $\dot{V}O_{2\max}$, with 2‐min rest between bouts), *R* (RQ) values decreased and remained low for 3 h after exercise. Tucker et al. (2016) also reported that RQ (R) values observed after a sprint‐interval exercise significantly decreased and remained lower than those observed after moderate‐intensity prolonged exercise. In the present investigation, R (RQ) values observed from the end of the HIIE to 12:00 (~1.5 h after HIIE) were significantly lower than those observed on the control day, suggesting more fat oxidation after HIIE for the resting energy requirement. During HIIE that produces massive lactate, *R* exceed 1.0; low *R* (<0.70) observed immediately after such high‐intensity exercise are thought to be due to CO_2_ retention in response to the high‐intensity exercise (perhaps to replenish bicarbonate stores used to buffer the lactic acid produced). Blood lactate concentration remained significantly higher up to 1 h after HIIIE (T. Shimizu, K. Tsuji, Y. Xu, and I. Tabata, unpubl. observation), while blood lactate concentration at 1.5 h after HIIE was not different from the pre‐exercise value. We therefore reasoned that the *R* (RQ) values obtained in the present investigation may be underestimations due to mild hypocapnia related to blood lactate concentration that return to pre‐exercise values. It follows that the calculated amount of fat oxidation using *R* (RQ) observed in the present investigation might also be overestimated. RQ values after lunch and supper on the HIIE day did not differ from control‐day values. This result is similar to that reported by Laforgia et al. (1997), while average RQ values from 12:00–23:00 on the HIIE day (0.886 ± 0.020) were significantly less than those observed on the control day (0.907 ± 0.016) (*P* < 0.05), suggesting that carbohydrate oxidation stimulated by the meal might not have been enhanced further by prior HIIE. However, since we did not measure changes in glycogen concentration in skeletal muscle, it is not possible to conclude whether the further increase in meal‐stimulated oxygen consumption after HIIE should be attributed to oxygen uptake for synthesizing glycogen or/and for increasing metabolism stimulated by enhanced sympathetic nerve activity.

In summary, the present investigation demonstrated that HIIE significantly enhances the diet‐induced oxygen uptake, and that this enhancement is related to cardiorespiratory fitness.

## Conflict of Interest

The authors have no conflicts of interest to report. The results of the study are presented clearly, honestly, and without fabrication or inappropriate data manipulation.
